# Development of Home-Based Frailty Detection Device Using Wireless Sensor Networks

**DOI:** 10.1007/s40846-016-0127-y

**Published:** 2016-04-16

**Authors:** Chung-Chih Lin, Chun-Chang Chen, Pay-Shin Lin, Ren-Guey Lee, Jing-Siang Huang, Tsai-Hsuan Tsai, Yu-Chuan Chang

**Affiliations:** Department of Computer Science and Information Engineering, Healthy Aging Research Center, College of Engineering, Chang Gung University, 259 Wen-Hwa 1st Road, Kwei-Shan, Tao-Yuan, 333 Taiwan, ROC; Department of Graduate Institute of Computer and Communication Engineering, National Taipei University of Technology, 1 Chung-hsiao E. Rd. Sec. 3, Taipei, 10608 Taiwan, ROC; Department of Physical Therapy, Chang Gung University, 259 Wen-Hwa 1st Road, Kwei-Shan, Tao-Yuan, 333 Taiwan, ROC; Department of Industrial Design, Chang Gung University, 259 Wen-Hwa 1st Road, Kwei-Shan, Tao-Yuan, 333 Taiwan, ROC

**Keywords:** Frailty, Wireless sensor technology, Degradation

## Abstract

This study develops a home-based frailty detection device that uses embedded systems and wireless sensing technology. This system helps monitor the impact of aging among the elderly through wireless automatic detection. The detection system consists of four devices. The first device, called eScale, simulates the traditional falling ruler test to measure reaction time. Another device, called eChair, measures the pressure exerted by a test subject through a pressure sensor. It is used to test three symptoms of frailty, namely slowness of movement, physical weakness, and body weight. The third device, called ePad, consists of a soft membrane switch placed on the ground to detect footsteps and is used to test balance. The fourth device, called eReach, measures displacement through ultrasound sensors. It is used to carry out the functional reach test. The sampling rate of each device is the main factor that determines system performance. When the test distance was set to 5 m for Home-Gateway, a 1-Hz sampling rate showed the best performance (98 %). Up to eight devices can be connected simultaneously to the gateway. The proposed system was compared with conventional approaches through testing with test subjects (n = 8). The results of the five tests were as follows: standing forward bend (r = 0.929), balance (r = 0.996), slowness of movement (r = 0.976), and physical weakness (r = 0.991), with *p* < 0.01. In the reaction time test, r = 0.871, with *p* < 0.1. All results suggest high correlations. Tests of aging symptoms were performed on 309 people aged over 65 years. Among males, degradation of over 20 % was found in the areas of physical weakness, slowness of movement, and functional reach. Among females, a degradation of 75 % was found in the balance test.

## Introduction

The symptoms of frailty are commonly seen in the elderly population. Clinically, frailty is defined as the decrease in physiological reserves and resistance to pressure due to cumulative physiological functional degradation [1]. Normal frailty causes a decrease in physiological reserves in many systems. Serious frailty results in a rapid deterioration of overall health. In developed countries, among people aged over 65 years, about 10–25 % show signs of frailty. Frailty can be controlled and eased [2, 3]. However, due to the limited understanding of frailty, and the lack of methods for the long-term tracking of health, it is often not possible to prevent frailty and other aging problems among the elderly.

Clinically, there is a lack of standards for measuring frailty, making it difficult to define frailty and diagnose it. Based on existing literature [4–9], this study defines the following four types of frailty: physiological frailty, psychological frailty, sociological frailty, and functional frailty. Functional frailty can be detected through subjectively defined symptoms such as physical exhaustion and low physical activity [10]. It can also be measured objectively through devices monitoring weight loss, physical weakness, slowness of movement, balance, functional reach, and reaction time. Traditional measurement methods require a medical practitioner to perform tests on a person and record data according to procedure. Subjects cannot perform the tests on themselves. This is not convenient for the long-term recording and monitoring of health status and frailty. In view of this limitation, the present study proposes a wireless home-based frailty detection system. It can easily be used to detect frailty at home with the help of wireless communication technology. In comparison to traditional clinical tests, the test procedure is simplified. All the testing parameters of clinical tests are measured, allowing people to monitor symptoms of frailty as part of their day-to-day lives.

## Materials and Methods

### Traditional Frailty Measurement Methods

The traditional test of weakness is 30 s of sitting and standing [11]. Assistants are required to help track the time and record the number of sit and stand motions. The slowness of movement test measures the 3-m walking time [12]. The balance test requires the subject to close their eyes and stand on one foot, with the assistant recording the posture holding time [13]. Functional reach measures the maximum distance an individual can reach forward while standing in a fixed position [14]. The reaction time is a measure of how long it takes a person to react to a falling ruler.

### Home-Base Frailty Detection Device Design

**Fig. 1:**
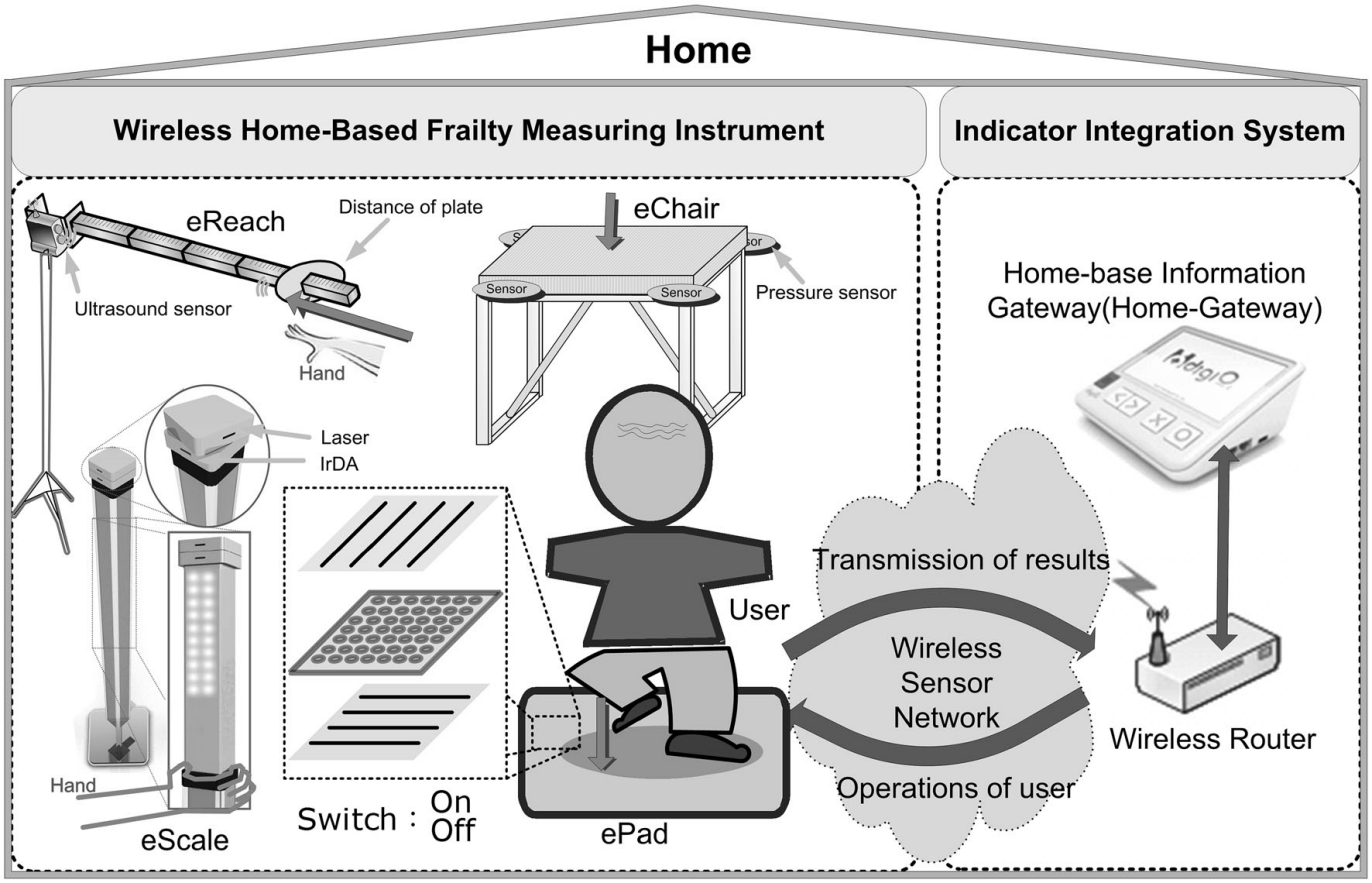
Home-based wireless frailty detection system

This study, based on traditional clinical measurement tools and methodology, developed the eScale, ePad, eChair, and eReach wireless home-based frailty detection devices. The architecture of the home-based wireless frailty detection system is shown in Fig. 1. It consists of hardware devices with an integrated measurement system. There are four wireless hardware devices for frailty detection. The integrated measurement system consists of wireless routers and the Home-Gateway. Wireless routers receive physical data transmitted by all devices and upload the frailty data to the Home-Gateway, which processes and records the data for long-term analysis.

### eScale Design for Reaction Time and Slowness Measurements

**Fig. 2:**
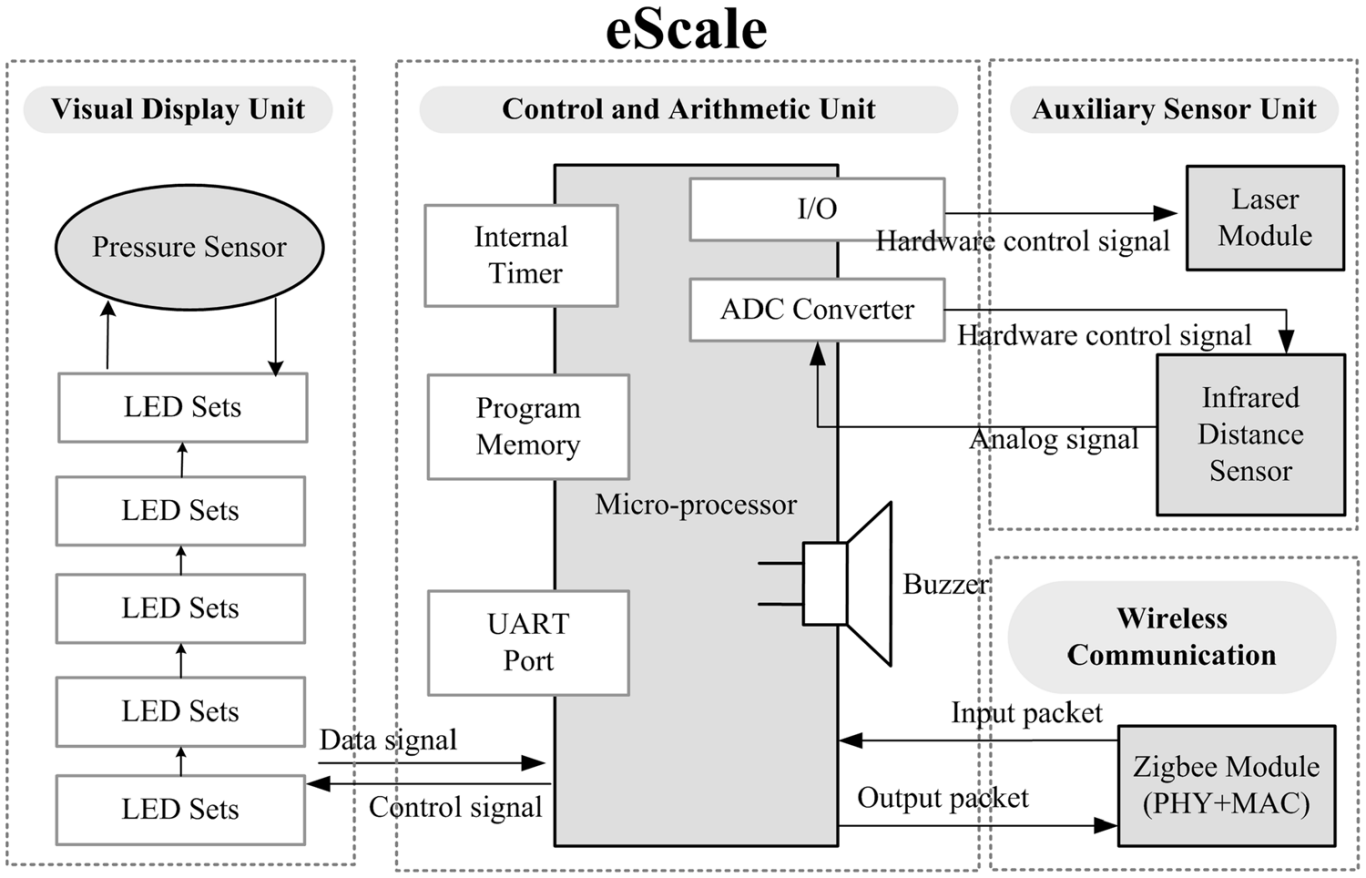
Function block diagram of eScale

LEDs are used to show the position of eScale. When eScale falls, the device automatically records the time the subject takes to touch the coiled pressure sensor. The time is then uploaded to the Home-Gateway, as shown in Fig. 2. The device hardware consists of the display unit, the control unit, the wireless communication module, and the sensor unit. The display unit used for this study uses several LEDs to simulate the visual effect of traditional methods. The LEDs are turned on from the top to the bottom in order to simulate a falling ruler. The subject’s reaction is monitored by the pressure sensor unit through the subject’s finger. The display unit consists of several 8-piece LED lights and the pressure sensor. The length of the ruler can be adjusted by increasing or decreasing the number of 8-piece LED lights. The 8-piece LED lights are connected in series, with a total length of over 80 cm. In the beginning, LEDs in the top 50 cm section are turned on. The column of lit LEDs simulates the current position of the ruler. In this state, the “ruler” is ready and can be released at any time. The falling movement of the ruler is controlled by the LED shifter, which lights up LEDs from top to bottom sequentially to visually simulate a falling ruler. The LED display time period determines the falling speed. The less time each LED is turned on for, the faster is the falling speed. The falling speed is consistent with the free fall speed. The time and distance of free fall are related as:

1 $$S = \frac{1}{2} \times gt{}^{2}$$

where *S* is the total length of the electronic ruler, *g* is acceleration due to gravity (=9.8 m/s^2^), and *t* is the falling time. The time taken for the ruler to fall to each LED position is different. This equation is used to pre-compute the reach time, i.e., the time when an LED position is reached during free fall. The display time of an LED is the difference between the reach times, i.e., the reach time of the current LED minus the reach time of the previous LED. The display time of each LED position is computed to control the falling speed and simulate free fall. The material used for the coiled pressure sensor is polyester. Its linearity (error) is < ±5 % and its lag is < ±4.5 % FSR (Full Scale Range). It is used to detect pressure exerted by the test subject. When the button is pressed, a signal is sent to the microprocessor. The device communicates with a computer using a Zigbee wireless transmission module, which forwards the received packets to the Home-Gateway. In the reaction time test, eScale provides a laser module. The laser is pointed towards the ground to measure the 3-m range. Furthermore, eScale can also be used with eChair to perform the 3-m up & go test, and it contains an infrared sensor to check whether the subject has passed the 3-m range, which is signaled back to the control unit.

### eChair Design for Pressure Measurement

**Fig. 3:**
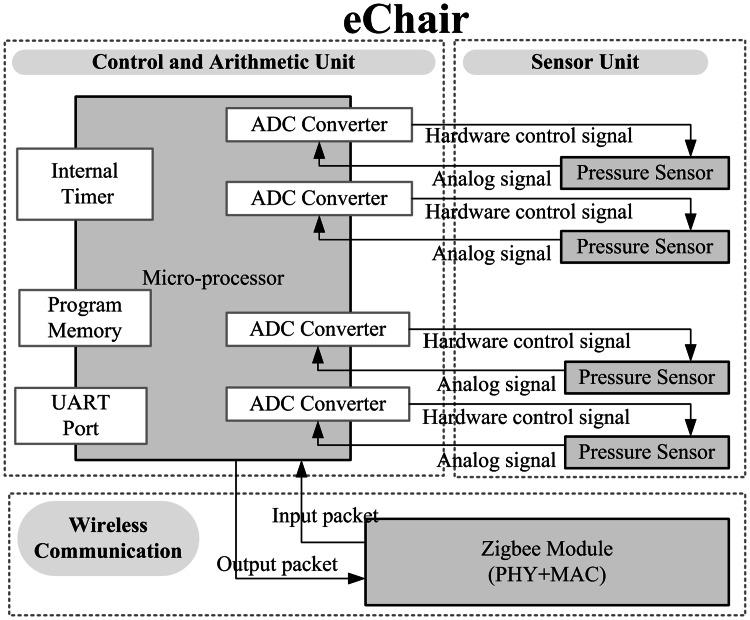
Function block diagram of eChair

eChair is capable of sensing changes in pressure, and thus can be used to measure body weight, weakness in the 30-s sit & stand test, and slowness of movement in the 3-m up and go test. eChair comprises a control and arithmetic unit, a sensor unit, and a wireless communication module. The hardware architecture of eChair is shown in Fig. 3. The control and arithmetic unit judges sensor signals, exchanges data control packets, executes various control programs, and provides timer and prompt tone functions. The unit controls various operation functions of instrument and components. For the sensor unit, the weight sensors should be provided with pressure scales, and pressure sensors are selected for the sensor component. Four sensors are installed at four corners of the scale. Under a certain stress, the the sensor’s structure undergoes tiny changes. These tiny changes alter the sensor’s output voltage. Therefore, the voltage variation can be converted to weight. For structural design, the electronic scale has a major metal support frame to bear weight. The major support frame is four-sided, and pressure sensors are installed at the four vertex corners of the support frame. The pressure sensor measures the pressure exerted by the support frame to the floor, and sends back the pressure variation. Each pressure sensor can bear a weight of 30 kg, and the whole structure of eChair can bear a weight of approximately 120 kg. The range of eChair covers the weight of normal elders. The wireless communication module is a low-power Zigbee wireless transmission module. The module is responsible for exchanging messages and packets with the Home-Gateway.

### ePad Design for Balance Measurement

**Fig. 4:**
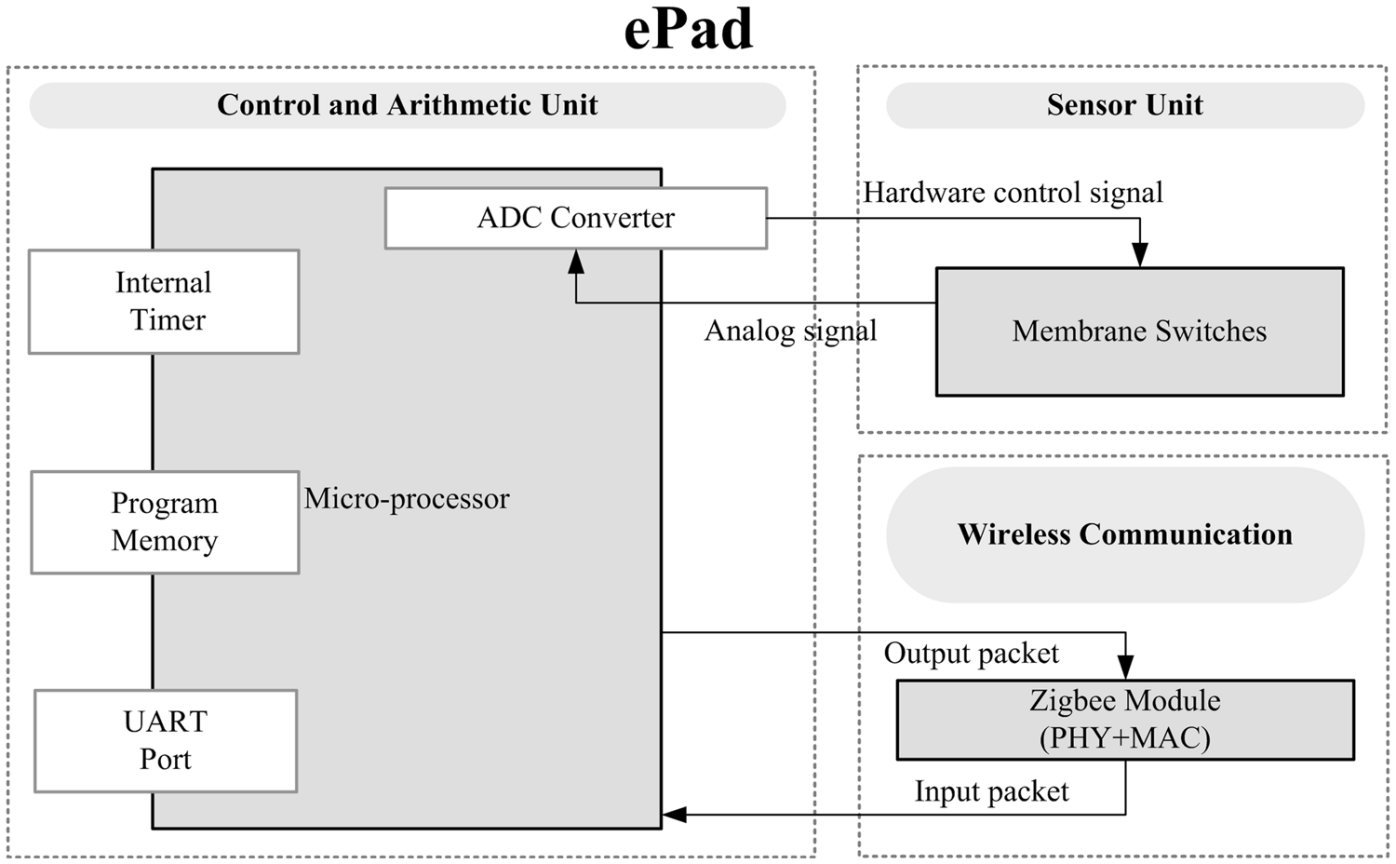
Function block diagram of ePad

ePad measures balance in the closed-eye balance test. Since the test requires room for movement, the step pad needs to be large. Therefore, a soft membrane switch is used as the sensor unit. The soft membrane switch consists of two layers of conductive film separated by a layer of insulation foam. The insulation foam has holes (φ 10 mm) at a fixed distance from each other. The hardware circuit diagram of ePad is shown in Fig. 4. When the sensor is pressed by a test subject, the conductive films connect through the holes. The connectivity of the films reflects the subject’s steps. The control unit is responsible for converting the signals and encoding and decoding the communication packets. Through the Zigbee transmission module, the balance signals are uploaded to the Home-Gateway.

### eReach Design for Functional Reach Measurement

**Fig. 5:**
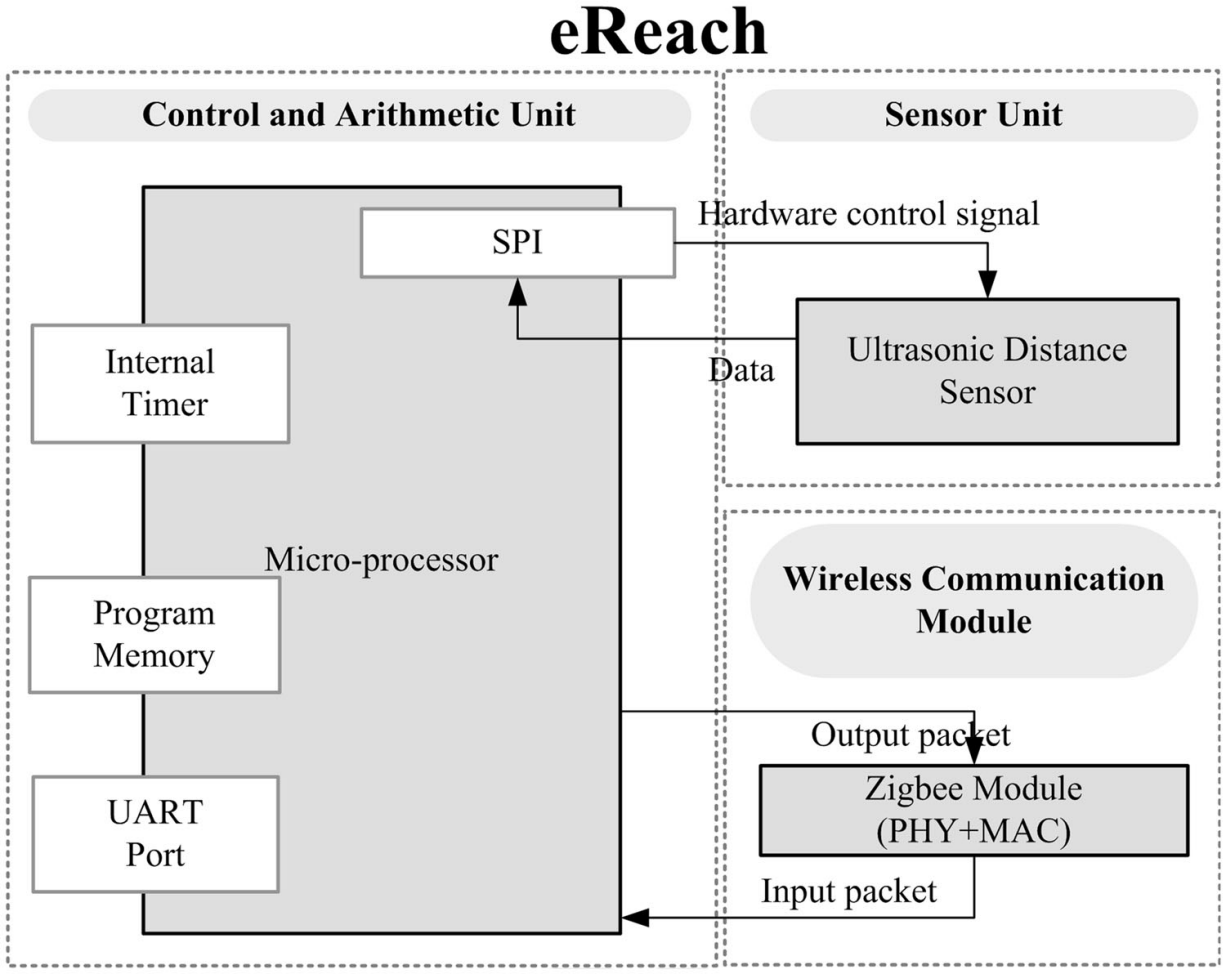
Function block diagram of eReach

This device tests functional reach. For this test, a subject performs a standing forward bend. eReach has an ultrasound distance sensor and control module. The wiring diagram is shown in Fig. 5. The device consists of a microcontroller unit (MCU), a distance sensor, and a wireless communication module. The MCU uses the SPI interface to read the ultrasound sensor’s measurement. The ultrasound sensor is installed at the bottom of a conventional measurement instrument, and monitors distance changes of activity and event. The maximum sampling frequency of the sensor is 10 Hz, which is sufficient for collecting a user’s body forward bending distance variation. The MCU transmits the distance measurement to the Home-Gateway using the Zigbee wireless communication module. The ultrasound distance sensor used in this study has a detection range of 3–60 cm, with a resolution of 1 mm. The frequency of the ultrasound is 40 Hz. The control unit calculates the distance of plate when object bend. A higher value indicates the better flexibility.

### Communication Packet Design and Communication Protocol

In this paper, wireless communication, sensors, and an embedded system were combined for use in a home environment. The proposed system for personal healthcare and elderly care can reduce the cost of medical care.

**Fig. 6:**
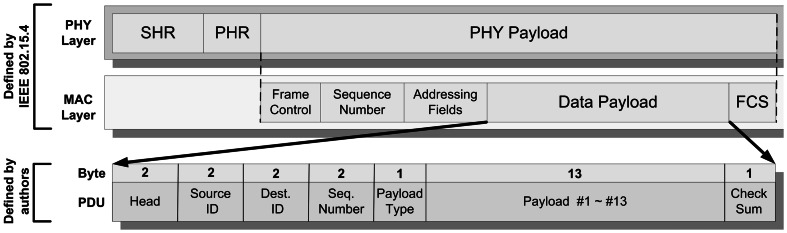
Packet format of communication protocol

**Fig. 7:**
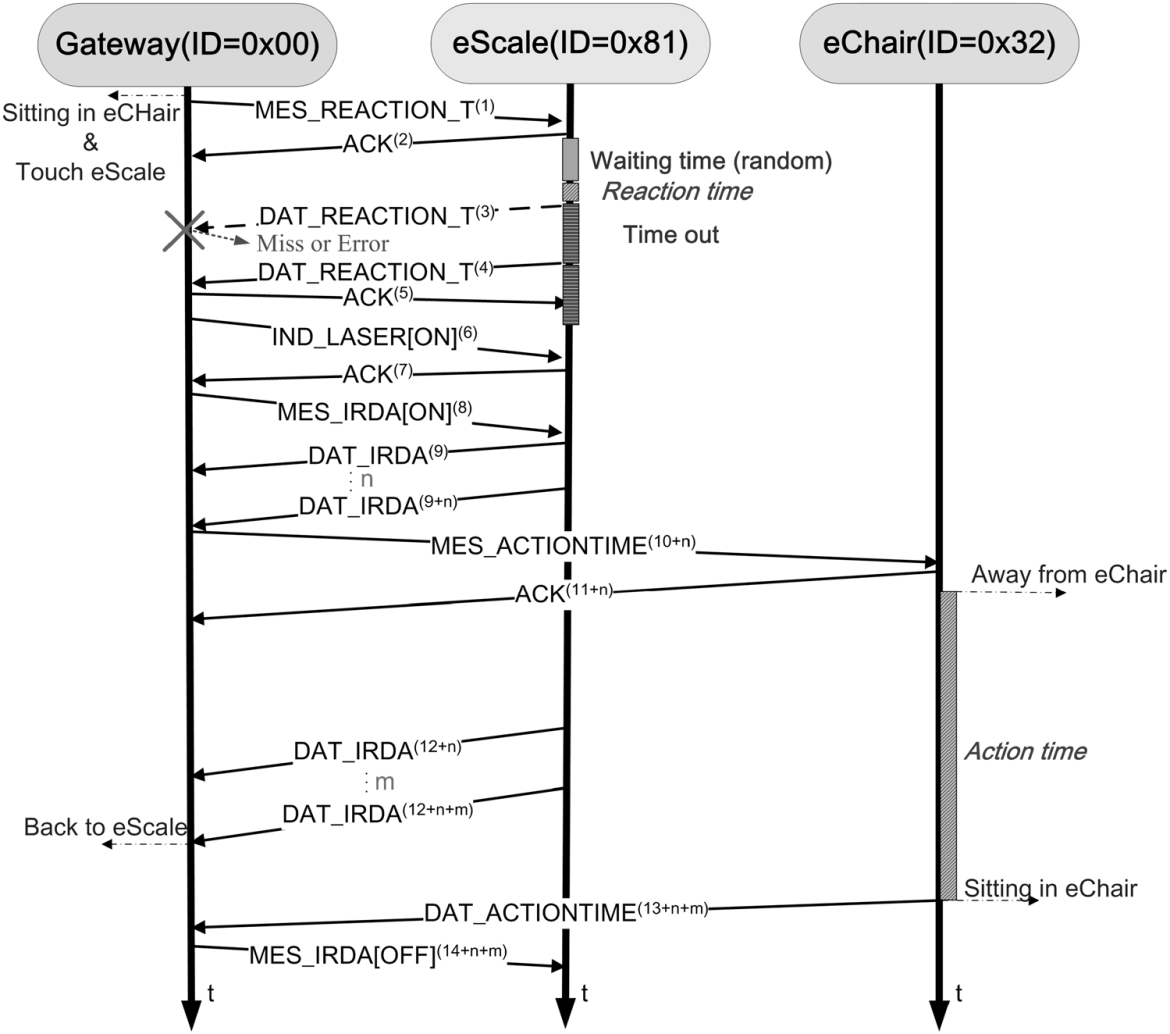
Time chart for communication of devices (measurement of reaction time and slowness). *Note* Message_Name^(n)^ indicates n-th transmitted Message_Name packet

IEEE 802.15.4 is a standard that specifies the physical layer and media access control for low-rate wireless personal area networks. It is a simple but flexible package data communication protocol, and can meet ordinary service quality requirements. IEEE 802.15.4 offers an optimized design for applications with a low data transmission rate. It offers longer battery service life, a flexible network architecture, and low-complexity hardware and software design schemes [15–23]. The IEEE 802.15.4 communication protocol is thus used by the wireless communication modules for communication between the frailty detection devices and the Home-Gateway (as shown in Fig. 6). The packet format used in this system is an extension of the MAC Data Payload field format. The total length of each packet is 23 bytes. Each packet includes two fixed headers, source ID (the identifier for the source device, representing its category and number), dest. ID (the identifier of the destination device, representing its category and number), Seq. Number (sequence number of the packet), and Payload Type (type of packet, can be instruction type and data type, including Payload #1 ~ #13). Figure 7 shows the state diagram for the communication of the devices. When eScale alone is used to test reaction time, the Home-Gateway first sends the “MES_REACTION_T” instruction packet. eScale receives this instruction and responds with an ACK packet to the Gateway, indicating that it has received the instruction to test reaction time. Then, eScale waits a random amount of time, and begins the LED light sequence to simulate the falling ruler to test the subject’s response time. After the test is finished, it returns “DAT_REACTION_T” data to the Gateway. If the packet is lost or contains errors, eScale waits for a certain amount of time (timeout) and sends the packet again. When the Gateway has received the “DAT_REACTION_T” packet, it sends back an ACK packet to eScale, indicating that it has received the correct packet for reaction time.

The eScale and eChair devices are used together to perform the 3-m up & go test for slowness of movement. When eScale receives the “IND_LASER[ON]” packet from the Home-Gateway, it turns on its laser to indicate the designated 3-m region on the ground. Then, Gateway sends a “MES_IRDA[ON]” packet to turn on infrared distance detection, which determines whether a subject has left the eScale 3-m region. Then, the Gateway sends the instruction “MES_ACTIONTIME” to eChair to calculate the total time spent on movement in the 3-m region. The above procedure involves the use of eScale and eChair, or any other devices to perform frailty test; this makes it much easier for subjects to perform the test at home.

## Results and Discussion

The cases used in this study were collected from volunteers in Chang Gung Hospital and Rehabilitation Center. The degeneration information of 309 people (178 female, 131 male) aged over 65 years was collected in this study. Four experiments were conducted to test the overall system performance: (1) effective transmission distance, (2) limitation of sampling rate, (3) reliability and validity test, and (4) establishment of clinical profiles.

### Effective Transmission Distance

**Fig. 8:**
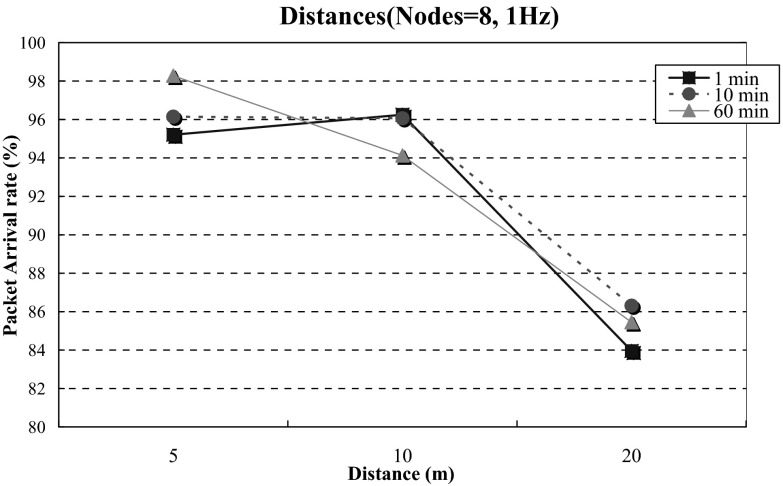
Effective transmission distance

In the transmission distance experiment, the distances from the devices to the Home-Gateway were 5, 10, and 20 m, respectively. The test periods were 1, 10, and 60 min, respectively. Devices sent packets at a fixed frequency of 1 Hz. Eight devices were used. Experimental results are shown in Fig. 8. As the distance increases, system performance degrades significantly. For example, in the 60-min experiment, when the distance is 5 m, the transmission rate is 98.28 %. When the distance is increased to 20 m, the rate drops to 85.45 % on average. When the distance between the device and Home-Gateway increases, the transmission signal strength becomes weaker, and the package arrival rate declines [24–27].

### Limitation of Sampling Rate

The variables used in the sampling rate experiments were the transmission frequency of the devices and the test distance. The system sent out data packets at frequencies of 1, 10, and 50 Hz, respectively. The test distance was 5 m. Eight devices were tested for 1 min. The sampling rate drastically affects the processing and transmission capability of the system. When the sampling rate was 1 Hz, the system’s processing efficiency was 95.21 %. At 10 Hz, it was 67.65 %, and at 50 Hz, it was 25.47 %. When the sampling rate of a device increases, the transmitted data size increases, making the package arrival rate decrease [28, 29].

### Maximum Carrying Capacity of Measurement Devices

**Fig. 9:**
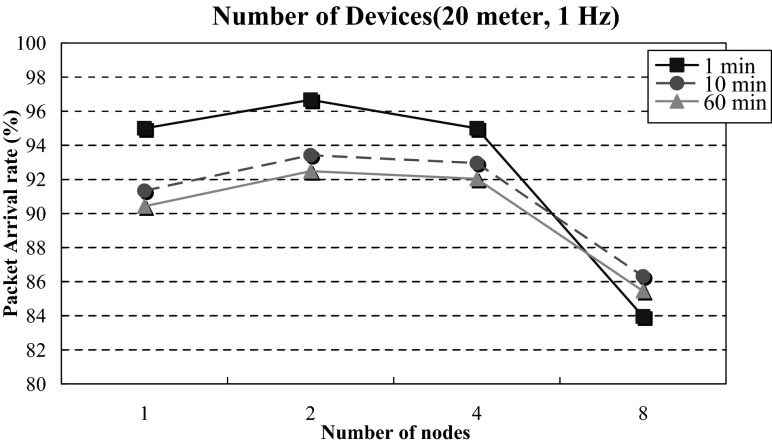
Maximum carrying capacity of measurement devices

The test variables of the carrying capacity experiment were device number and test time. The numbers of measurement devices were 1, 2, 4, and 8, respectively. The test times were 1, 10, and 60 min, respectively. The measurement device sent test packets at a fixed frequency of 1 Hz. The distance between Home-Gateway and the measurement device was 20 m. Figure 9 shows the test results. The worst outcome occurred when the test time was 60 min and the device number was 8, with a package processing ratio of 85.45 %. It can be observed that under a long-time test, increasing the number of measurement devices slightly affects transmission and processing capabilities. Two sets of measurement devices (2 electronic measurement scales, 2 electronic mats, 2 electronic scales, and 2 forward bending measurement devices) installed in a home environment can operate normally for a long-time test. When the number of devices increases, the data size of the whole network increases, making the package arrival rate decrease [30–32].

### Reliability and Validity Test

In order to verify that the electronic devices offer the same level of reliability and validity as those of traditional tools, five frailty measures, namely reaction time, balance, functional reach, physical weakness, and slowness of movement, were tested. Eight individuals were tested. The Pearson correlation coefficients are listed in Table 1. The falling ruler test (reaction time) has a correlation coefficient of r = 0.871, *p* < 0.01. The closed-eye foot balance test (balance ability) has a correlation of r = 0.996, *p* < 0.001. The standing forward bend (functional reach) test has a correlation of r = 0.929, *p* < 0.001. The 30-s sit & stand test (physical weakness) has a correlation of r = 0.991, *p* < 0.001. The 3-m up & go test (slowness of movement) has a correlation of r = 0.976, *p* < 0.001 × 10^−5^. The reaction time test has the lowest correlation (r = 0.871, *p* < 0.01).

**Table 1:** Correlations of frailty detection device

	Measurement method	Mean	Standard deviation	Pearson correlation
Functional reach	Traditional test	33.75	5.94018	0.929**
	Proposed system	34.9957	4.73616	
Reaction time	Traditional test	19.5	3.02372	0.871*
	Proposed system	21.4125	3.36131	
Balance	Traditional test	28.9563	7.22315	0.996**
	Proposed system	28.725	7.09079	
Slowness of movement	Traditional test	5.8838	1.05481	0.976**
	Proposed system	6.0125	1.12686	
Weakness	Traditional test	22.25	5.77556	0.991**
	Proposed system	21.75	5.65054	

* *p* < 0.01, ** *p* < 0.001, N = 8

### Establishment of Clinical Profiles

According to the World Health Organization (WHO), an aging society is defined as a society in which the number of people aged over 65 years surpasses 7 % of the total population. Studies have shown that people aged over 65 years will have rapid aging of their body functions and use more medical resources. In Europe and the United States, people aged over 65 years account for 10–25 % of the total population [33]. Therefore, this age group was selected as the degradation indicator reference standard. Using the system introduced in this paper, frailty profiles were collected for 309 people aged over 65 years. The average age was 76 years. The data were aggregated and analyzed to establish the references for frailty measures in each age and gender group, as shown in Tables 2 and 3. In a given age group, males perform better than females for all measures. Reaction time tests showed that, with an increase in age, the falling distance of the LEDs increased from 33.17 to 35.78 cm for males, and from 39.26 to 40.1 cm for females. The degradation of reaction time in females is not as much as that in males. The physical weakness measure of males declined 32.2 %, from 18.29 to 12.4 times. For females, it declined about 50.7 %, from 16.64 to 8.19 times. For slowness of movement, males showed a decline in speed of 20.8 %, from 0.72 to 0.57 m/s. For females, the speed decreased by 28.4 %, from 0.67 to 0.48 m/s. The time that males were able to maintain balance decreased from 4.97 to 3.28 s, whereas that for females decreased from 5.54 to 1.37 s. This decrease in females of about 75 % is much higher than that in males. In the functional reach test, the performance among males dropped 27.5 %, from 32.06 to 23.23 cm, whereas that among females dropped 18.3 %, from 26.55 to 21.69 cm.

**Table 2:** References for frailty measures in each age group for males

Males	Frailty measures				
Age group (years)	Reaction time (cm)	Weakness (times)	Slowness of movement (m/s)	Balance (s)	Functional reach (cm)
65–69	33.17	18.29	0.72	4.97	32.06
70–74	33.82	16.82	0.68	4.54	29.85
75–79	34.47	15.35	0.64	4.12	27.64
80–84	35.13	13.88	0.60	3.70	25.43
85–89	35.78	12.40	0.57	3.28	23.23

**Table 3:** References for frailty measures in each age group for females

Females	Frailty measures				
Age group (years)	Reaction time (cm)	Weakness (times)	Slowness of movement (m/s)	Balance (s)	Functional reach (cm)
65–69	39.26	16.64	0.67	5.54	26.55
70–74	39.47	14.53	0.63	5.50	25.33
75–79	39.68	12.42	0.58	3.45	24.12
80–84	39.89	10.31	0.53	2.41	22.90
85–89	40.1	8.19	0.48	1.37	21.69

## Conclusion

This study developed a home-based frailty detection device that uses embedded systems and wireless sensing technology. The proposed detection devices simplify the test procedure for frailty. Two sets of measurement devices (2 electronic measurement scales, 2 electronic mats, 2 electronic scales, and 2 forward bending measuring devices) installed in a home environment can operate normally for a long-time test. Test results show an over 20 % decline in physical weakness, slowness of movement, and functional reach. Among females, a remarkable 75 % reduction in balance was found. It increased with age and was greater in woman than in man. Through long-term monitoring, it is possible to analyze and monitor frailty and collect data from more users. This data can provide personalized exercise suggestions to decrease degradation and increase the quality of life. In the future, this can make the evaluation of new medical treatments possible, and can help improve the lives of the elderly.
